# Application of the *AMPLE* cluster-and-truncate approach to NMR structures for molecular replacement

**DOI:** 10.1107/S0907444913018453

**Published:** 2013-10-12

**Authors:** Jaclyn Bibby, Ronan M. Keegan, Olga Mayans, Martyn D. Winn, Daniel J. Rigden

**Affiliations:** a Institute of Integrative Biology, University of Liverpool, Liverpool L69 7ZB, England; b Research Complex at Harwell, STFC Rutherford Appleton Laboratory, Didcot OX11 0FA, England; c Science and Technology Facilities Council Daresbury Laboratory, Warrington WA4 4AD, England

**Keywords:** molecular replacement, *AMPLE*, NMR structures, search models

## Abstract

*AMPLE* is a program developed for clustering and truncating *ab initio* protein structure predictions into search models for molecular replacement. Here, it is shown that its core cluster-and-truncate methods also work well for processing NMR ensembles into search models. *Rosetta* remodelling helps to extend success to NMR structures bearing low sequence identity or high structural divergence from the target protein. Potential future routes to improved performance are considered and practical, general guidelines on using *AMPLE* are provided.

## Abstract 2

Processing of NMR structures for molecular replacement by *AMPLE* works well.

## 1. Introduction

Molecular replacement (MR) is an increasingly common route to solving the phase problem for protein crystal structures. In 2012, for example, 77% of protein structures submitted to the Protein Data Bank (PDB; Rose *et al.*, 2012 ▶) were solved using MR. MR involves the placement of an existing structure (the search model) in the new unit cell of the target structure in such a way as to reproduce its crystallographic lattice. This provides approximate phasing information allowing the initial calculation of electron-density maps (Rossmann & Blow, 1962 ▶). Typically, the search model is derived from an experimental structure. The structure itself, a processed version of it or a homology model of a related protein may all be used, but the existence of a similar structure to the target is ultimately required.

Historically, NMR structures have been considered to be more problematic than crystal structures for use as search models in MR: it is not uncommon for a crystal structure to be insoluble even with an NMR structure of the same protein (Chen *et al.*, 2000 ▶). While genuine conformational differences may exist between the crystalline and solution states of a protein, more frequently the problem stems from the intrinsic variability within NMR ensembles and the fact that NMR structures generally score more poorly by protein structure-quality measures than their crystal structure counterparts (Bhattacharya *et al.*, 2007 ▶). In NMR, spectral overlap and peak broadening are factors that reduce the number of experimental restraints that can be assigned to specific parts of the molecule and hence employed in model calculation. The reduced experimental definition of the affected areas translates into their increased conformational variability during structure calculation and thus results in local divergence within the NMR ensemble (Doreleijers, Sousa da Silva *et al.*, 2012 ▶).

Nevertheless, the introduction of residual dipolar couplings (RDCs; Tjandra & Bax, 1997 ▶) has provided valuable long-range information that helps to define large-scale features of the protein structure, and general progress in NMR methods has led to a corresponding improvement in the quality of NMR structures (Mao *et al.*, 2011 ▶).

Irrespective of methodology, some protein regions may be truly mobile, lacking defined structure, and hence more variable in calculated NMR ensembles. Such locally divergent regions in NMR ensembles tend to deviate more strongly from the crystal structure counterpart. Thus, in order to reduce noise in the MR search model, variable regions in NMR ensembles are normally excluded. Since the most variable regions in an NMR ensemble are commonly surface loops and termini that broadly correlate with regions of higher *B* factors in crystallographic structures, their elimination often has the additional advantage of avoiding some less well defined parts in the crystal structure, *i.e.* those that contribute least to the scattering. Recently, a specialized tool, *FindCore* (Snyder & Montelione, 2005 ▶), has been applied to processing NMR ensembles for MR (Mao *et al.*, 2011 ▶). *FindCore* reduces variability within the NMR ensemble by calculating an atomic pseudo-*B* factor based on structural variance and eliminating any atoms (plus trailing side-chain atoms) with a pseudo-*B* factor of over 60 Å^2^. In a benchmarking exercise using *Phaser* for MR and using *ARP*/*wARP* for tracing, *FindCore*-derived ensembles solved 22 of 25 cases in which the NMR ensemble and the target crystal structure were 100% sequence-identical, and a further two cases were successful when *Rosetta* refinement was employed (Mao *et al.*, 2011 ▶). In cases of homologous proteins a sequence-identity threshold was observed: above 40% identity success was assured, but below 30% identity only one of four cases yielded a correct solution.

As stated, among the predicted structures used for MR, homology models predominate. However, in recent times there has been rapid development in the area of *ab initio* protein modelling (also known as *de novo* or template-free modelling; Gajda *et al.*, 2012 ▶). This aims to predict protein structures without relying on evolutionary relationships and so can address novel folds that are inaccessible to homology modelling. Although the combinatorial nature of the *ab initio* folding algorithm limits the accessible target size to around 120 residues for soluble proteins or 145 residues for membrane proteins (Yarov-Yarovoy *et al.*, 2006 ▶; Barth *et al.*, 2007 ▶), *ab initio* models have successfully been employed for MR. This was first with performed compute-intensive all-atom models (Qian *et al.*, 2007 ▶; Das & Baker, 2009 ▶). More recently, more cheaply obtained predictions have been employed (Rigden *et al.*, 2008 ▶; Bibby *et al.*, 2012 ▶) using a cluster-and-truncate approach combined with different modes of side-chain treatment. Now implemented as the *CCP*4 program *AMPLE*, the pipeline produces many search models for each target (up to around 500) and succeeds over a range of search-model size from very small, generally accurately modelled structures to larger more approximate representations.

As mentioned above, locally divergent regions in NMR ensembles often differ most from the corresponding crystal structures and are often eliminated prior to MR attempts. This is conceptually similar to the rational elimination of divergent and likely inaccurate regions by *AMPLE* in ensembles derived from *ab initio* modelling (Bibby *et al.*, 2012 ▶). We therefore explored the application of *AMPLE* to NMR ensembles, proposing too that its sampling of both large and small search models, combined with different side-chain treatments, could improve performance compared with the approach of finding a single core structure (Mao *et al.*, 2011 ▶). Here, we describe the results, demonstrating the successful application of *AMPLE* to solve crystal structures using search models derived from NMR structures. Furthermore, we find that a protocol including *Rosetta* (Leaver-Fay *et al.*, 2011 ▶) remodelling of NMR structures can lead to successful structure solution where simple editing does not. Detailed hands-on guidance for running *AMPLE* is also provided (see Appendix *A* [App appa]).

## 2. Materials and methods

### 2.1. Materials

For comparison with previous results, we assessed the performance of *AMPLE* against the set of 25 matching (100% sequence-identical) NMR search models and target crystal structures, recently solved, previously used with the *FindCore* method of search-model preparation (Mao *et al.*, 2011 ▶). Additionally, we used a set of ten NMR ensembles of thio­redoxin-fold proteins to try to solve the crystal structure of *Streptomyces coelicolor* thioredoxin (PDB entry 1t00; 112 residues; diffraction data to 1.51 Å resolution; Stefankova *et al.*, 2005 ▶) both with *AMPLE* and *FindCore*. Thioredoxin proteins were chosen as providing a broad range of sequence identities *versus* the target from 16 to 52% (Table 1 ▶), allowing a better definition of the limits of success. NMR ensembles were assessed for structural quality using the *NRG-CING* server (Doreleijers, Sousa da Silva *et al.*, 2012 ▶), resulting in an ROG overall molecular classification (Doreleijers, Vranken *et al.*, 2012 ▶) of red (lower quality), orange (intermediate) or green (higher).

### 2.2. Methods

NMR ensembles were processed into search models by *AMPLE* in two ways. The first treats the NMR ensemble in the same way as described previously for processing a set of *ab initio* models (Bibby *et al.*, 2012 ▶). Briefly, *AMPLE* determines the conformational diversity of C^α^ atoms in the NMR ensemble along the protein chain using *THESEUS* (Theobald & Wuttke, 2006 ▶). This guides the truncation of the NMR ensemble in 5% steps starting with the most variable regions and with application to whole residues. The set of truncated ensembles are subclustered at different radii and subjected to three modes of side-chain treatment: retention of all side chains, elimination of all side chains beyond C^β^ or retention of only a subset. The subset are those that the side-chain prediction program *SCWRL* (Canutescu *et al.*, 2003 ▶; Krivov *et al.*, 2009 ▶) places most accurately, a consideration that is not relevant to the processing of NMR models but is related indirectly to side-chain conformation variability in a way that might help to preferentially eliminate ill-defined surface residues. Alternative side-chain treatments oriented specifically towards NMR ensembles, *e.g* elimination according to conformational variability, will be explored in the future. Processing an NMR ensemble into a set of search models typically takes around 15 min. The resulting set is then passed to *MrBUMP* (Keegan & Winn, 2008 ▶) for MR with both *Phaser* (McCoy *et al.*, 2007 ▶) and *MOLREP* (Vagin & Teplyakov, 2010 ▶). The resulting top placements are then treated to rapid phase modification and C^α^ tracing in *SHELXE* (Usón *et al.*, 2007 ▶; Sheldrick, 2010 ▶): resulting CC scores of >25 are reliably indicative of correct placement and often result from near-complete automatic tracing of the structure (Rodríguez *et al.*, 2012 ▶). Thus, a CC score of >25 was our stringent measure of the success of a given search model.

Where simple truncation as above failed to give a correct solution, additional processing of the NMR ensemble with *Rosetta* was tried. This is based on previous observations that the phasing power of NMR ensemble-derived search models can be improved by *Rosetta* (Qian *et al.*, 2007 ▶). Our refinement consisted of an initial idealization of each model of the NMR ensemble using the *idealize* application of *Rosetta* (Leaver-Fay *et al.*, 2011 ▶) followed by comparative modelling and relaxation using the *mr_protocols* application (DiMaio *et al.*, 2011 ▶). In the present work, no electron density is provided to this application. The comparative modelling protocol was applied using the sequence of the NMR structure separately to each member of the ensemble. 1000 models were generated, sampling each member of the NMR ensemble equally. Since the number of conformers in each deposited NMR ensemble varies, the number of times that each conformer is used as the basis for remodelling will vary. A typical run time for generating the 1000-model set is 13 h, making it comparable in overall timing to similarly sized *ab initio* modelling cases. This set of models was then treated in the same way as the sets of 1000 decoys generated *ab initio* previously (Bibby *et al.*, 2012 ▶).

For comparison, *FindCore* was also applied to the thio­redoxin test set. For each NMR ensemble, *FindCore* indicated a list of core residues. Non-core residues were removed and the result was used for MR and rebuilding. This was performed with *AMPLE* invoking the -ensembles flag without any further modification. Structural superpositions were performed with *TM-align* (Zhang & Skolnick, 2005 ▶).

## 3. Results

### 3.1. Sequence-identical NMR ensembles and target crystal structures

Previous work had shown a good success rate (22 from 25) using the *FindCore* program to prepare MR search models from NMR ensembles sharing 100% sequence identity (Mao *et al.*, 2011 ▶). In the same work, *Rosetta* refinement of the NMR ensemble prior to MR solved a further two cases. As Supplementary Table S1^1^ shows, *AMPLE*, with and without *Rosetta* remodelling, performs similarly well with this test set. Truncation alone in *AMPLE* solves 19 of 22 successes of *FindCore* to the point of automatic tracing in *SHELXE*, while *Rosetta* remodelling leads to success for the same additional two cases. In three cases previously successfully solved by *FindCore* the *AMPLE* pipeline failed: for these, diffraction data to only 2.4–2.5 Å resolution were available, which is at the limit of the range in which *SHELXE* is reliable.

### 3.2. Thioredoxin-fold test cases of non-sequence-identical NMR models

**Figure 1:**
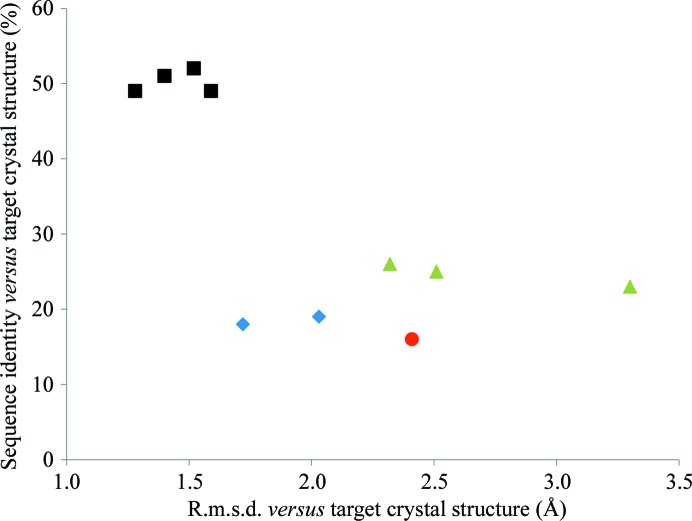
Results of attempts to solve the crystal structure of *S. coelicolor* thioredoxin (PDB code 1t00) with various NMR structures using *FindCore* and *AMPLE* for search-model preparation. Squares indicate success with both programs, diamonds indicate *AMPLE*-only successes with a truncation-only protocol, triangles indicate *AMPLE*-only successes with a *Rosetta* remodelling protocol and the circle indicates a case that was not solved with either program.

The successes of *FindCore* and *AMPLE* on a set of thio­redoxin-fold NMR structures with various levels of sequence and structural similarity to a selected crystallographic target are shown in Table 1 ▶ and Fig. 1 ▶. Four cases (the easiest, with sequence identities of >49% and r.m.s.d. values of <1.6 Å) were solved with both programs. Straightforward truncation of the NMR ensembles was successful with *AMPLE* alone for two cases with very low sequence identity (<20%) but moderate structural conservation (1.7–2.1 Å r.m.s.d.). An additional remodelling step prior to the clustering and truncation protocol of *AMPLE* allowed the solution of three cases that were around 25% sequence-identical to the target but ranged widely in their structural difference from it. These include a case in which the r.m.s.d. was very high at 3.30 Å. One case, PDB entry 2b5x (Zhang *et al.*, 2006 ▶), with the lowest sequence identity (16%) was not soluble, even with the remodelling. Interestingly, this ensemble contained only 11 conformers, the fewest among the NMR structures used: it remains to be seen whether this relative lack of sampling of structural space contributed to its failure.

**Figure 2:**
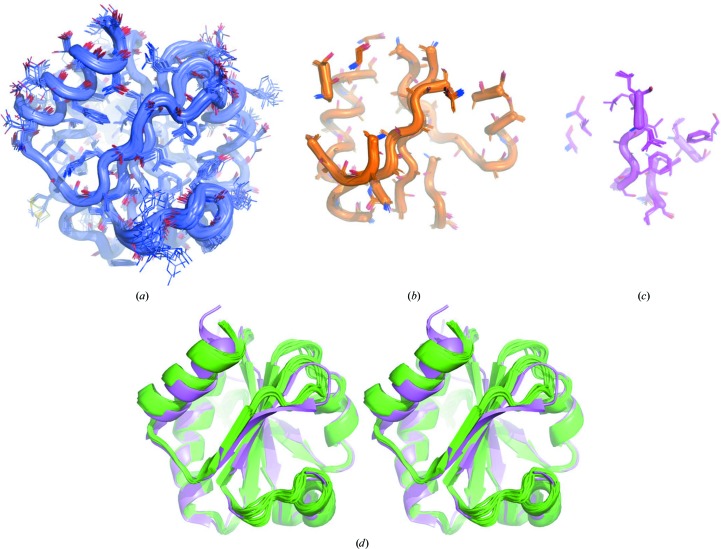
*AMPLE* processes NMR structures into successful search models of various sizes. The structure of *E. coli* thioredoxin (PDB entry 1xoa; Jeng *et al.*, 1994 ▶) yields successful search models to solve the crystal structure of *S. coelicolor* thioredoxin (PDB entry 1t00) containing, for example, (*a*) 108 residues (untruncated) retaining all side chains, (*b*) 60 residues with side chains trimmed to C^β^ and (*c*) 15 residues with only selected side chains retained. A stereo comparison of the 1xoa ensemble (green) and the target crystal structure, *S. coelicolor* thioredoxin (PDB entry 1t00; magenta), is shown in (*d*). The figure was produced using *PyMOL* (http://www.pymol.org).

**Figure 3:**
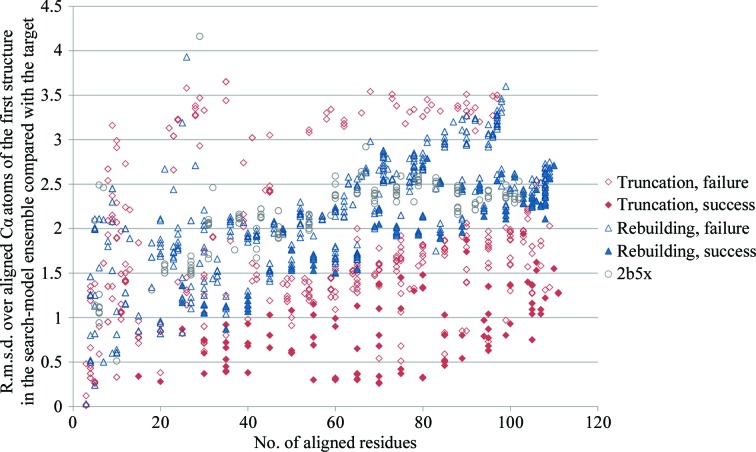
Success and failure of structure solution as a function of search-model size and r.m.s.d. difference from the target. Structure comparisons were performed with *TM-align* (Zhang & Skolnick, 2005 ▶), aligning a majority of the structures with a mean ten residues of the search model left unaligned. Search models relating to cases solved by simple truncation are shown as red diamonds and those relating to cases solved by rebuilding are shown as blue triangles. Filled symbols indicate successful search models. Grey circles indicate search models for PDB entry 2b5x, the single unsolved case.

As described previously (Bibby *et al.*, 2012 ▶), successful *AMPLE*-derived ensembles ranged broadly in size (Table 1 ▶; Figs. 2 ▶ and 3 ▶). The smallest, derived from *Escherichia coli* thioredoxin, contained 15 residues, which was only 14% of the NMR structure. Below this, presumably, even extremely accurate search models contain too little phasing information for success. The largest was 133 residues from human protein disulfide-isomerase A6 domain 2. Also as described previously, there is a correlation between the r.m.s.d. of the search model *versus* the target and successful search-model size (Fig. 3 ▶): for both the simple truncations and the remodelling cases a larger r.m.s.d. is tolerated for larger search models, whereas smaller search models must be more accurate for success.

## 4. Discussion

We tested the cluster-and-truncate methods of *AMPLE* on NMR structures even though they were specifically developed and optimized to process a very different type of structure: *ab initio* protein models. The comparison with recent work using the *FindCore* program to process NMR ensembles is illustrative (Mao *et al.*, 2011 ▶). With a set of sequence-identical test cases performance is very similar, but a current limitation of *AMPLE* leads to failure in three cases that were solved with *FindCore*. We ascribe this to the resolution of the data available in these cases of 2.4–2.5 Å, which is at the limit of the capabilities of *SHELXE*. Thus, although *SHELXE* is a very powerful and convenient tool, particularly for its ability to distinguish correct MR solutions using a reliable statistic, it can constrain the success of *AMPLE* as a whole in some cases. As well as its resolution limits, its much better performance with α-helical proteins compared with all-β proteins must also be borne in mind. Future development of *AMPLE* will allow a case-dependent choice of rebuilding tool.

The performance of *AMPLE* in the thioredoxin test set was very encouraging, solving cases with low sequence identity (18%) and/or high structural divergence from the target (3.3 Å r.m.s.d.). Although based on a single fold and calling for further confirmation, these results compare very well with *FindCore*, which only solved the thioredoxin structure with NMR structures of >49% sequence identity. This is in line with previous *FindCore* results, in which structures with >40% sequence identity were solved routinely but those with <30% sequence identity were solved only rarely (Mao *et al.*, 2011 ▶). The broad positive correlation seen in Fig. 3 ▶ between search model-to-target r.m.s.d. and number of aligned residues suggests that the more divergent regions targeted by truncation in *AMPLE* are generally those that differ most between the available NMR structure and the target crystal structure and hence those that are the most advantageous to remove. It is likely that more extensive sampling also contributes to the additional success of *AMPLE*. Applied to *ab initio* models, *AMPLE* can generate up to 500 or so search models per case. The numbers were smaller here since *AMPLE* processes the three largest clusters of *ab initio* models while the NMR ensemble was treated here as a single cluster: the number of search models per case here ranged from 183 to 213. The benefits of sampling a range of sizes and side-chain treatments are graphically illustrated by the single success obtained using the tryparedoxin NMR structure (PDB entry 1okd; Krumme *et al.*, 2003 ▶; Table 1 ▶). The unique successful search model was 105 residues long and had all side chains cropped back to C^β^.

Taken as a whole, the results from the application of *AMPLE* to NMR structures are already very promising and suggest that it is a useful alternative to *FindCore* or manual processing. In particular, there are clear suggestions that it can extend success to harder cases of lower sequence identity and structural similarity between NMR structure and target crystal structure (Table 1 ▶, Fig. 1 ▶). Encouragingly, there are obvious possibilities to improve the performance further. At present, the side-chain methods in *AMPLE* are tailored to the *ab initio* model scenario: an explicit consideration of side-chain variability in the NMR ensemble would allow a better treatment in the resultant search models. For example, only those side chains that are experimentally poorly defined could be eliminated. Such protocols will be implemented in future versions of *AMPLE*.

We used NMR ensemble validation (Doreleijers, Sousa da Silva *et al.*, 2012 ▶) to assess whether structural quality could be limiting MR performance in some cases. The validation, based on residue-level stereochemical analyses, results in molecule-level quality ROG ratings of red (lower), orange (intermediate) or green (better). Although the numbers are too small to draw firm conclusions, there are hints that red-rated or orange-rated ensembles are less prone to solve crystal structures straightforwardly. In the comparison with *FindCore* (Supplementary Table S1), most NMR ensembles are of high structural quality (green) and can typically be solved, without *Rosetta* refinement, using either *FindCore* or *AMPLE*. Of the two ‘red’ ensembles, one solves straightforwardly and the other requires *Rosetta* rebuilding for success with both *FindCore* and *AMPLE*. The single case that does not solve with either program, even with *Rosetta* rebuilding, is ‘orange’. The thioredoxin cases (Table 1 ▶) are harder to interpret since the percentage sequence identity between NMR ensembles and crystal structure varies, but within the nine *AMPLE* successes two of the three in which *Rosetta* rebuilding was required are ‘orange’, whereas only one ‘orange’ ensemble solved the target without rebuilding. If confirmed, this suggests that future improvements in NMR methodology and consequently ensemble quality would feed through into improved performance in MR. Also interestingly, the single structure that failed to solve (PDB entry 2b5x), although ‘green’, contains a minimized average structure in its ensemble, a practice that is now deprecated, and is the only structure in the set to do so.

In conclusion, we have previously shown that the cluster-and-truncate methodology is an effective tool for processing *ab initio* models, and in the current article we have shown that it is also powerful for processing NMR ensembles. This central idea can also be applied to other scenarios, and we are currently investigating its use in completing partial MR solutions and its application to specific structural classes such as transmembrane domains and coiled-coil proteins.

## Supplementary Material

Supplementary material file. DOI: 10.1107/S0907444913018453/kw5070sup1.pdf

## Figures and Tables

**Table 1:** Thioredoxin-fold NMR structures used for MR search-model preparation tests with *FindCore* and *AMPLE* against the target structure *S. coelicolor* thioredoxin (PDB entry 1t00; 112 residues; diffraction data to 1.51 Å resolution) Ensembles were classified for structural quality using *CING* validation as green (better), orange (intermediate) or red (worse). Structures are ordered by decreasing sequence identity *versus* the target crystal structure.

PDB code of NMR structure	Protein	Length (residues)	Sequence identity *versus* target 1t00 (%)	ROG class from *CING* validation	C^α^ r.m.s. deviation (Å) of first member of ensemble *versus* target 1t00, No. of atoms matched	Solved with *FindCore*?	Size of *FindCore* search model (residues)	Solved with *AMPLE* by truncation?	Solved with *AMPLE* by remodelling?	Size range of successful *AMPLE* search models (residues)	C^α^ r.m.s. deviation (Å) range of first members of successful *AMPLE* search models *versus* target 1t00
1xoa	*Escherichia coli* thioredoxin	108	52	Green	1.52, 108	Yes	58	Yes	—	15–108	0.28–1.22
1dby	*Chlamydomonas reinhardtii* thioredoxin M	107	51	Green	1.40, 107	Yes	67	Yes	—	25–107	0.46–1.48
2gzy	*Bacillus subtilis* thioredoxin	104	49	Green	1.28, 104	Yes	59	Yes	—	55–104	0.93–1.40
2l4q	*Mycobacterium tuberculosis* thioredoxin C	116	49	Green	1.59, 110	Yes	61	Yes	—	30–115	0.26–1.55
1x5d	Human protein disulfide-isomerase A6 domain 2	133	26	Green	2.32, 109	No	79	No	Yes	84–133	2.11–2.62
2diz	Human thioredoxin domain-containing protein 5, domain 3	117	25	Orange	2.51, 107	No	74	No	Yes	25–117	0.87–2.71
1okd	Tryparedoxin	154	23	Orange	3.30, 96	No	95	No	Yes	105 alone	2.79
2l6c	*Desulfovibrio vulgaris* desulfothioredoxin	110	19	Green	2.03, 103	No	55	Yes	—	90–104	1.74–1.94
2diy	Human thioredoxin domain-containing protein 2, thioredoxin domain	130	18	Orange	1.72, 108	No	89	Yes	—	78–108	1.30–1.62
2b5x	*Bacillus subtilis* YkuV thiol-disulfide oxidoreductase	148	16	Green	2.41, 102	No	105	No	No	—	—

## Footnotes

1 Supplementary material has been deposited in the IUCr electronic archive (Reference: KW5070). Services for accessing this material are described at the back of the journal.

## Appendix A Using the *AMPLE* software

*AMPLE* has several modes of operation. As well as allowing the user to have some control over its procedures, these different options allow different approaches to solving a particular molecular-replacement problem. Some of this functionality is exposed in the *CCP*4*i* interface to *AMPLE*, but the full range of options is only available from the command line. Here, we give an overview of the protocols in *AMPLE* along with a brief description of how to interpret the output of the program. For detailed user documentation on *AMPLE*, the reader is directed to the *CCP*4 wiki site at http://ccp4wiki.org.

### A1. Basic procedure

The primary function of the program is to create or receive as input *ab initio* models (‘decoys’) and to prepare them for use as MR search models. Currently, the program can use the *Rosetta* package to produce these decoys given the target sequence. The decoys are assembled from fragments that can be derived locally if additional programs and databases are installed (Gront *et al.*, 2011 ▶). Alternatively, fragments can be obtained from the *Robetta* web server (Kim *et al.*, 2004 ▶; http://www.robetta.org) and supplied to *AMPLE*. Decoy models can also be obtained by the user from other programs such as *QUARK* (Xu & Zhang, 2012 ▶). *AMPLE* accepts these from the user and subjects them to the MR search-model preparation procedures. In all cases, the user must provide the structure-factor amplitudes in the form of an MTZ file for use in the MR step.

### A2. Accepting and remodelling externally provided structures

In order to allow maximum flexibility, *AMPLE* can be directed to retrieve user-provided models from a given directory. This allows *AMPLE* to work with NMR ensembles, as described above, but also to accept models that the user may have obtained in other ways. Prior to the generation of search models, *AMPLE* can be directed to carry out *Rosetta* remodelling. Using a fragment-based technique, *Rosetta* can repeatedly remodel and refine an input structure to produce an ensemble of structures that can be dealt with by clustering and truncation.

### A3. Missing domains

As mentioned above, *AMPLE* can be used to create search models for locating missing domains in cases where one or more components have already been found. This procedure can take advantage of the information provided by the existing model regarding the separation of the termini of the missing domain. *Rosetta* uses this information to restrain the distance between the termini of the decoy models. The user specifies the value for this distance along with the model for the already known part of the target structure.

### A4. Interpreting the *AMPLE* output

The MR preparation procedures in *AMPLE* can create hundreds of search models. In many of our test cases, such a broad sampling of parameter space was necessary since only a small number of combinations of clustering, truncation and side-chain treatment produced successful search models. As a result, running *AMPLE* can take several days of CPU time on a single machine. It is possible to speed up the processing by taking advantage of multiple cores or by submitting the underlying decoy-generation and MR steps to a compute cluster (currently, Sun Grid Engine is supported). An early indication of the likelihood of success is given by the results of the decoy-clustering step and this is reported by *AMPLE*. A large top cluster consisting of many more decoys than the subsequent clusters is indicative of potentially more accurate *ab initio* modelling, which can be expected to result in more successful search models. Once the program enters the molecular-replacement stage, the user is presented with a summarized table of the results for each of the search models that have completed their processing. Refinement statistics along with CC scores from *SHELXE* (where used) are presented and ordered according to the final *R*_free_ value after 30 cycles of restrained refinement of the MR solution in *REFMAC* (Murshudov *et al.*, 2011 ▶).

The field of *ab initio* modelling is developing rapidly: improvements in accuracy (Xu & Zhang, 2012 ▶) and, excitingly, in the size of the target that can potentially be addressed (see, for example, Karakaş *et al.*, 2012 ▶) have recently been made. In addition, developments in the area of molecular-replacement, phase-improvement and model-building software continue apace. We anticipate that these developments will help *AMPLE* to become an increasingly valuable tool in structure solution and make it applicable to an ever-broadening range of target structures. However, users of *AMPLE* should be aware of the current limitations of the program. To date, we have only been able to determine structures with experimental data to 2.5 Å resolution or better and the *ab initio* protocol is highly unlikely to work on targets that are longer than around 130 residues since this is the upper size limit accessible to the modelling.
