# Supplementary material for: Application of the AMPLE cluster-and-truncate approach to NMR structures for molecular replacement
Source: Acta Crystallogr D Biol Crystallogr. 2013 Oct 12;69(Pt 11):2194–201. doi: 10.1107/S0907444913018453 (PMC3817692; doi:10.1107/S0907444913018453)
Supplement: Supplementary file 1 [file d-69-02194-sup1.pdf]

Supplementary Table S1: Comparison of FindCore and AMPLE performance on a set of paired, sequence-identical crystal and NMR structures (Mao *et al.*, 2011). The NMR ensembles were classified for structural quality using CING validation (Doreleijers *et al.*, 2012) as green (better), orange (intermediate) or red (worse).

Target	PDB code of crystal structure	Length (residues)	PDB code of NMR ensemble	ROG class from CING validation	Solved with FindCore ?	Solved with FindCore and ROSETTA refinement ?	Solved with AMPLE by truncation	Solved with AMPLE and ROSETTA refinement ?
BeR31	3cpk	150	2k2e	green	Yes	-	Yes	
CcR55	2o0q	115	2jqn	green	Yes	-	Yes	
CsR4	2ota	76	2jr2	green	Yes	-	Yes	
CtR107	3e0h	158	2kcu	green	Yes	-	Yes	
CtR148A	3ibw	88	2ko1	green	Yes	-	Yes	
GmR137	3cwi	78	2k5p	green	Yes	-	Yes	
HR1958	1tvg	153	1xpw	green	Yes	-	Yes	
HR3646E	3fia	121	2khn	green	Yes	-	Yes	
MbR242E	3gw2	108	2kko	green	Yes	-	Yes	
MrR110B	3e0e	97	2k5v	green	Yes	-	Yes	
OR8C	2rhk	140	2kkz	green	Yes	-	Yes	
PfR193A	3idu	127	2kl6	green	Yes	-	Yes	
SgR42	3c4s	66	2jz2	green	Yes	-	Yes	
SoR77	2qti	80	2juw	green	Yes	-	Yes	
SR213	2im8	131	2hfi	green	Yes	-	Yes	
SR384	3bhp	60	2jvd	green	Yes	-	Yes	
SsR10	2q00	129	2jpu	green	Yes	-	Yes	
StR65f	2es9	115	2jn8	green	Yes	-	Yes	
XcR50	1ttz	87	1xpv	red	Yes	-	Yes	
HR41	3evx	175	2k07	green	Yes	-	No*	
PsR293	3h9x	125	2kfp	green	Yes	-	No*	
StR70	2es7	142	2jzt	green	Yes	-	No*	
DrR147D	3ggn	155	2kcz	orange	No	No	No	No
SR478	2gsv	80	2js1	green	No	Yes	No	Yes
ZR18	2ffm	91	1pqx	red	No	Yes	No	Yes

* The limited resolution of diffraction data (2.4-2.5 Å) for these cases means failure at the SHELXE step at the end of AMPLE is the likely explanation.

Doreleijers, J. F., Vranken, W. F., Schulte, C., Markley, J. L., Ulrich, E. L., Vriend, G. & Vuister, G. W. (2012). *Nucleic Acids Res.* **40**, D519-24.
Mao, B., Guan, R. & Montelione, G. T. (2011). *Structure.* **19,** 757-766.
